# Advanced paternal age: effects on sperm parameters, assisted reproduction outcomes and offspring health

**DOI:** 10.1186/s12958-020-00668-y

**Published:** 2020-11-13

**Authors:** Iman Halvaei, Julia Litzky, Navid Esfandiari

**Affiliations:** 1 Department of Anatomical Sciences, Faculty of Medical Sciences, Tarbiat Modares University, Tehran, Iran; 2 Department of Pediatrics, Dartmouth Hitchcock Medical Center, Lebanon, NH USA; 3 Department of Obstetrics, Gynecology and Reproductive Sciences, University of Vermont Medical Center, Larner College of Medicine, 111 Colchester Ave, Burlington, VT 05401 USA

**Keywords:** Male age, Aging, Pregnancy, Live birth, Sperm

## Abstract

Many factors, including postponement of marriage, increased life expectancy, and improved success with assisted reproductive technologies have been contributing to increased paternal age in developed nations. This increased average paternal age has led to concerns about adverse effects of advanced paternal age on sperm quality, assisted reproductive outcomes, and the health of the offspring conceived by older fathers. This review discusses the association between advanced paternal age and sperm parameters, assisted reproduction success rates, and offspring health.

## Introduction

Recent reports from the US have shown an increase in average paternal age of 3.5 years over a 44 year period, with this increase being consistent across all races, ethnicities, and regions, and regardless of level of education [65]. Similar trends have been seen in several European countries [94]. Many factors have been found to be contributing to this trend, including postponement of marriage, increased life expectancy, increased financial capital, increased tendency to delay starting families to pursue postgraduate and higher education and find a suitable career, and improved contraceptive access. Additionally, increased rates of divorce and remarriage, resulting in many fathers having a child with a second spouse or partner later in life, has contributed to increasing paternal age. Improved methodologies for assisted reproductive technologies (ARTs) have paralleled these lifestyle and cultural changes, allowing for couples to start families later in life despite declining fertility [14]; introduction of intrauterine insemination (IUI), in vitro fertilization (IVF), and intracytoplasmic sperm injection (ICSI) has allowed for childbearing in males with reduced fertility due to advanced age.

Although many of the challenges of conception and longer-term outcomes, including aneuploidy, are attributable to maternal age, increasing evidence is showing that advanced paternal age (APA) may also have negative effects on gamete genetics, conception rates, miscarriage and live birth rates, and long-term health of offspring. Studies into these topics, however, have been limited by a lack of clear definition of APA, with ages from 35 to 45 commonly used [63, 97]. In addition, separating the effects of APA from those of advanced maternal age (AMA) are complicated by the collinearity of maternal age with paternal age, as most older males are partnered to older women; One study found a 70% correlation between maternal and paternal ages [110]. Several studies have used oocyte donor cycles to avoid this complication, but these studies are challenged by small sample sizes. As average paternal age increases in developed nations, it is becoming increasingly important that we understand the effects of APA so that couples can receive appropriate counseling and prognostics. In this paper, we will review the effects of APA on sperm parameters and infertility treatment outcomes, as well as long-term health of the offspring of APA men. Our main goal is to improve awareness regarding APA for both healthcare providers and for all patients to be informed about potential risk factors associated with APA so they can make informed reproductive choices.

### Semen characteristics

Increased paternal age is associated with significant decreases in several sperm parameters including semen volume and sperm count, motility, morphology, and viability. Although the direct cause of the relationship between these variables and male age is unknown, there are many potential mechanisms that are known to change with age that include decreased function of reproductive accessory glands, cellular and physiologic changes including a decrease in the capacity to repair cellular and tissue damage, decrease in germ cells and androgen levels, and structural changes to the male reproductive anatomy including narrowing the seminiferous tubules, vascular insufficiency, and systemic disease related to aging [9, 35, 54, 93]. In particular, accessory gland secretions are reduced in older men and the water and protein content are different from that in younger men, [66] which may affect sperm motility. Spermatogenesis may also be impaired in aging resulting in abnormal sperm morphology in older men [84]. A combination of all of these etiologies is likely responsible for the outcomes discussed below, but further research is needed to establish causal relationships.

#### Semen volume

Increasing male age has been found to be associated with decreased semen volume [62, 105], which is secondary to a decrease in accessory gland secretion [66]. Specifically, each year of age has been associated with a 0.03 mL decrease in volume in one study [66], with another study finding a decrease of 0.22 mL for every 5 years of age [7]. An inverse relationship between male age and semen volume was identified in a large cohort study, with the decline becoming apparent at an average age of 35.5 years [120]. However, a more recent study found that the decline did not begin until after 50 years of age [98].

#### Sperm count

Several studies have found an inverse relationship between male age and sperm count, [62, 84, 98, 120], with two recent studies finding a significant decrease in sperm count starting at ages 41 and 41.5 [98, 120]. Despite this decrease, several studies have found increased sperm concentration (total sperm count) with increasing age [7, 13, 120], likely due to the simultaneous decrease in semen volume with age [120]. One study reporting an increase of 3.1 × 10^6^ sperm/mL for every 5 years of age [7], with another study identifying an increase starting at age 30.5 years [120]. However, a systematic review and meta-analysis found that sperm count did not change with increasing age but did find that sperm concentration was decreased in older men, potentially due to a decreased semen volume [62]. Importantly, age was not found to be associated with sperm retrieval rate in testicular sperm extraction [99].

#### Sperm motility, morphology, and viability

Several studies have reported reduced sperm motility with increasing male age [7, 62, 120], with one study finding a 1.2% decrease for every 5 years of age [7], with just one reporting no change in motility with age [87]. Progressive motility, total motility and all sperm kinematics have been found to have negative correlations with increasing age [120]. In addition, Pino et al., found that the rate of progressive motility had a two-fold decrease in men > 50 in comparison with 40–50 year-old men [98]. However, there is little evidence for an association between sperm morphology and APA [62, 105], when studied continuously stratified between men < 40 years old and ≥ 40 years old [120], nor between three different age groups (31–40, 41–50 and >50) [98]. Sperm viability has been consistently found to decrease with aging [13, 84], with one study finding a decrease starting at age 41.5 and significant reductions in viability in men ≥40 compared to those < 40 years (83.75 ± 8.78 vs. 81.49 ± 10.60, respectively) [120].

#### Round cells and peroxidase-positive cells

There are also some conflicting data regarding the effect of APA on the number of round cells and peroxidase-positive cells in semen, with one study finding a decrease in round cells in men ≥40 compared to < 40 years but no difference in peroxidase-positive cells between these two groups [120], and another study found no significant differences in rates of either type of cells in men of the same age groups [101].

### Genetic changes in APA

As sperm quality decreases as men age, as discussed above, the integrity of sperm DNA also decreases. Although the direct cause of this change is unknown, these problems are likely related to the same etiologies as those affecting sperm characteristics, but especially impaired DNA repair and cell division regulation as men age. Additionally, risk of an accumulated damage from infection, alcohol, tobacco, and other toxins increases with age, and all of these likely affect the genetic integrity of sperm. As antioxidant capacity decreases with age [61], reactive oxygen species (ROS) may also increase with age, resulting in oxidative stress and DNA damage and increasing apoptosis in the testes [32, 54]. Again, a combination of factors is likely contributing to these outcomes, and additional research will hopefully provide improved understanding of the effects of age on sperm DNA.

#### Sperm DNA integrity

The importance of sperm DNA fragmentation to ART outcomes has been a well-debated topic, and although sperm DNA fragmentation has been associated with decreased rate of pregnancy in natural conception, IUI, and IVF and with increased rate of miscarriage [128], ASRM guidelines report insufficient evidence to verify the relationship between sperm DNA damage and ART outcomes. ASRM therefore recommends against the routine use of DNA integrity evaluation for infertility treatment, classifying DNA fragmentation testing as a Level C recommendation [82]. Given this potential relationship between ART outcomes and sperm DNA damage, several studies have explored the relationship between DNA fragmentation and paternal age, with most finding an increase in fragmentation with increasing age [46, 63]. Pino et al. found that in men > 50, the rate of DNA fragmentation index (DFI) is 4.58 times more likely to present compared to men < 30 years [98], and rates as high as ≥30% in men > 50, in comparison with 14.7% in men < 30 year old [85]. In a large study of 6881 semen samples, APA (> 45 years) was associated with higher DFI and lower high DNA stainability (HDS) than in the control group [29]. These findings may in part be due to a decrease in sperm apoptosis, as Singh et al. found an increase in DNA damage and decrease in sperm apoptosis in older men (cutoff: 35 years) [109]. Moskovtsev et al. found that those with high DNA damage (≥ 30%) also were more likely to have abnormal semen parameters [85].

A similar study by Das et al. found that an increase in DFI was associated with decreased sperm motility [24]. These findings have been consistent in both normozoospermic and oligoasthenoteratospermic patients in two studies [24, 101]. In contrast, Brahem et al. did not find increased rates of DNA fragmentation with increasing age in either an infertile or fertile control group, though their study was limited by a small sample size [13]. A separate prospective study evaluated 278 consecutive infertile patients undergoing their first IVF or ICSI treatment and similarly found no significant correlation between sperm DFI and male age [87].

#### Chromosomal aneuploidy

The relationship between AMA and chromosomal disorders, especially aneuploidy 21, has been well-established, and most studies have shown a similar association between increasing male age and increased rates of sperm and embryo aneuploidy and chromosomal aberration [13, 103]. The most common chromosomal aneuploidies in sperm have been found in chromosomes 21, 22, and X/Y, with about a 9% rate of chromosome abnormalities in spermatozoa, 7% of these being structural and 1–2% being numerical [76]. Although a recent meta-analysis found an association between APA (≥40 years) and trisomy 21 [91], only less than 10% of all trisomy 21 cases have been found to be due to paternal nondisjunction and spermatogenesis errors [107]. One important risk factor along with APA is advanced maternal age. It was shown that when maternal age was more than 35, the risk of trisomy 21 was increased [38].

##### In sperm

Griffin et al. evaluated about 400,000 spermatozoa from 24 men (aged 18–60 years) and found that the rate of XY, YY and XX disomy was positively associated with aging, suggesting that older men may be more likely to have offspring with aneuploidy than their younger counterparts [52]. Bosch et al. evaluated 190,117 spermatozoa from 18 healthy donors (aged 24–74 years) and found a linear increase in structural and numerical impairments in chromosome 9 with increasing age. They found a 29% increase in rates of chromosome 9 disomy, 18.8% increase in diploidy, and 14.6 to 28% increase in structural aberrations for every ten-year increase in male age [12]. Finally, a recent study has shown that the rate of sperm aneuploidy was significantly higher in men with advanced age (≥40 years) compared to younger men (14% vs. 4%, respectively) [63].

Increased rates of aneuploidy may be associated with arrested spermatogenesis. Dakouane et al., evaluated the histomorphometry of biopsied testis from 35 deceased donors (aged 61–102 years) using fluorescent in situ hybridization (FISH) to examine the X, Y and 18th chromosomes in these older men in comparison with 10 younger men aged 29–40 years. In older patients with normal spermatogenesis, rates of aneuploidy in post-meiotic cells were only 0.28% greater than the rates in the younger group (1.29% compared with 1.1%). However, older men with arrested spermatogenesis, as determined by thickening of the basal membrane, had a 14.28% aneuploidy rate [23]. However, in all of these studies, separating out the risk of aneuploidy associated with paternal age and the well-established risk associated with maternal age is extremely difficult, which may be further complicated by weaknesses in the FISH technique, especially in inability to detect nullisomy [39].

##### In embryos

Like sperm aneuploidy, there has been significant research into rates of aneuploidy in embryos conceived by older fathers, but with contradictory findings. Two studies by García-Ferreyra et al. have supported an increase in rates of aneuploidy in embryos conceived by older fathers. First, using 286 embryos obtained from 32 IVF/ICSI cycles using donor oocytes they found that the rate of aneuploidy from fathers of ≥50 years was significantly higher than the aneuploidy rate in younger fathers (< 50 years) [45]. In a separate study, also using embryos conceived with donor oocytes, these authors found significantly higher rates of aneuploidy 13, 18, and 21 in embryos fertilized with sperm from men > 50 years old in comparison with men 40–49 or ≤ 39 years [44]; rates for men ≥50 were over 14% for all three chromosomes, whereas the highest rate for the other age groups was 6.1% for chromosome 21 in men ≤39. In contrast, Carrasquillo et al. evaluated 6934 embryos from 1202 IVF/ICSI egg donor cycles using blastocyst biopsy and found no relationship between APA and embryo aneuploidy [18]. Similarly, a recent South Korean study found no association between paternal age and embryo aneuploidy, whereas significant differences were noted between groups that differed by maternal age [67]. The lack of consistent findings on APA and embryo aneuploidy is likely in part due to small sample sizes and differing methods of genetic testing. Although FISH has long been used for genetic testing, newer next generation sequencing techniques, combined with larger sample sizes, will hopefully be able to provide more accurate results that could better clarify the relationship [39].

### ART cycle outcomes

Pregnancy success rates following ART decline with increasing maternal age and significant research has been done on the relationship between AMA and the different ART treatment options. However, the ability to study APA and ART outcomes is often limited by data availability and difficulty to study in isolation as APA often occurs simultaneously with AMA. Nevertheless, many studies have found potential relationships between APA and poorer infertility treatment outcomes. These relationships are likely secondary to the above discussed increase in errors in spermiogenesis in the aged testes that result in reduced DNA quality [70] and increasing reactive oxygen species in semen [62], resulting in DNA damage, and potentially impairing activation of the male genome, which may increase risk of abortion. In a systematic review and meta-analysis by Zhao et al., the authors found that sperm DNA damage was associated with an increase in miscarriage following both IVF and ICSI and with a decreased pregnancy rate in ICSI cycles [128]. These findings supported those in a prior systematic review, which found lower pregnancy rates following natural conception, IUI, and IVF in those with sperm DNA damage, although they did not find such an association in ICSI-conceived pregnancies [129]. Findings on outcomes specific to each fertilization method are discussed below.

#### IUI outcome

In couples undergoing IUI, male age has been found to be a significant prognostic factor for pregnancy rate (female age: 28.5 ± 4.4 (18–40)) [50], even in couples with normal semen parameters and favorable female characteristics (female age: < 35) [30]. One multivariate analysis of 901 cycles found that the age of male (female age: 31.3 ± 4.5 (23–43)) was the most important factor affecting pregnancy rate in that cohort [77]. In one of the largest studies on this topic, Belloc et al. evaluated more than 17,000 IUI cycles and found that APA (> 45 years) was associated with poorer pregnancy rates and increased miscarriage rates [8]. In contrast, another study did not find an association between pregnancy loss and APA, nor with live birth rate (female age: 38.1 ± 4.00) [111].

Several recent studies have highlighted the difficulty in separating out APA from AMA. Tatsumi et al. analyzed 1576 IUI cycles in women under 40 years old and that paternal age alone, after adjusting for confounding factors, was not associated with pregnancy or live birth rates [113]. The adverse effects of paternal age on IUI outcomes were mostly attributable to maternal age. De Brucker and Tournaye reviewed the effect of female and male aging on IUI outcome and concluded that paternal age has synergistic negative effects on IUI success rate when the female partner is older than 35years [25]. Bellver et al. in a retrospective study evaluated 2204 IUI cycles and found no difference in pregnancy or miscarriage rates based on male age [10]. Given that men with APA are more likely to be partnered with women of AMA, evaluating the role of APA will likely continue to be challenging.

#### In vitro fertilization

ICSI is the fertilization method of choice for couples with paternal ages > 50 years [74], which is supported by improved outcomes using ICSI over IVF for older men; although fertilization rates for IVF and ICSI were not found to differ for men aged < 50, a higher fertilization rate was achieved using ICSI as compared to conventional IVF if the paternal age was > 50 [74]. When comparing fertilization rates for younger and older men for ICSI alone, however, several studies have found that older men have poorer fertilization rate outcomes following ICSI, with age group cut-offs of both ≥40 years and ≥ 50 years (65% vs. 56%, respectively, *P* = 0.02) [63] and [1], with one study finding a 0.3% decrease in fertilization rate for each additional year of paternal age [6].

These findings are supported by studies using donor oocytes, which remove the potential effects of concurrent AMA. Cito et al. found a strong negative correlation between paternal age and fertilization rate following ICSI in an oocyte donation program [21]. A second study using donor oocytes found that the fertilization rate in both conventional IVF and ICSI was inversely correlated with paternal age [74]. However, other studies of non-ICSI approaches have not provided as consistent data regarding fertilization rate and APA. A systematic review examining different ART types did not find a negative relationship between paternal age and fertilization rate [22]. Gu et al. evaluated 103 oocyte donation cycles from 70 couples (male aged 26–57 years) and found that fertilization rate in conventional IVF was not associated with paternal age [53]. Similarly, a retrospective study of 9991 IVF cycles found no relationship between paternal age and fertilization rate in different maternal age groups [126].

#### Embryo development and quality

Because sperm DNA may be associated with embryo development, the poorer quality of sperm DNA in APA may result in poorer embryo development outcomes. In addition, adverse effects of increasing male age on blastocyst formation may be related to impairment in male genome activation that occurs after cleavage-stage [22]. This is supported by studies that found no association between paternal age and cleavage-stage development [126], including in ICSI cycles with donor eggs [21]. In comparison with studies indicating poorer rates of blastocyst formation, lower quality embryos were available for cryopreservation at day 6 with greater paternal age (> 40 years) [74]. Several studies have looked at both embryo development stages in APA couples using donor oocytes. Frattarelli et al. evaluated 1023 men using donor oocytes and found that in men > 50 years, the rates of blastocyst formation were decreased. However, the rates of early embryo development, embryo arrest, cell number, and embryo cleavage were the same across age groups [42]. Similarly, Garcia-Ferreyra et al. evaluated 286 embryos obtained from 32 IVF/ICSI cycles using donor oocytes and found that, although the rate of good quality embryos on day 3 was not associated with paternal age, the rate of blastocyst development was decreased in cycles with men > 50 years [45]. APA was shown to have correlation with blastocyst formation rate, but not with the rate of top quality blastocysts [6]. In contrast to these findings, one recent study did find decreased rates of both embryo cleavage and blastocyst formation with APA (≥40 years) in comparison with younger men (97% vs. 94 and 33% vs. 24%, respectively) [63]; although this study did not use donor oocyte cycles, and so could not control for maternal age, all couples were being treated for male factor infertility and all women were < 40 years old.

#### Implantation rate

Studies of implantation rate in relation to APA face similar challenges regarding removing confounders, especially AMA, resulting in conflicting findings. Some authors have found an inverse relationship between paternal age and embryo implantation rates, with one study finding a 3.1% decrease in implantation rates for every additional year of paternal age, with significantly worse clinical pregnancy outcomes in men > 50 years old in comparison with men under 40 [56]. In cycles using donor oocytes, implantation rate was decreased when paternal age was > 38 years compared to ≤38 years (26.00 ± 1.52% vs. 32.43 ± 1.65%, respectively) [16]. In another study, when the paternal age was > 60 years, the implantation rate decreased from 43 to 22% when compared with cycles in men aged 50–59 years [74]. In contrast, no relationship between paternal age and implantation rate was found in several other studies [42, 63, 116], including in studies with donor oocytes [42]. In a retrospective study with a small sample size in which the authors used cryopreserved testicular spermatozoa in azoospermic men, there was no association between paternal age and implantation rate [116].

This variation in findings may be due to poor ability to control for confounding factors, as Capelouto et al. found that the small negative association they found between APA (> 35 years) and implantation rate was not observed after adjusting the confounding factors [17]. Sperm count may also be an important factor that is often not included as a potential confounder; Ferreira et al. found that paternal age negatively affects implantation rate in oligozoospermic patients undergoing ICSI cycles, whereas there was no correlation found between paternal age and implantation rate in normozoospermic patients [36]. Finally, maternal age may confound or modify the effects of APA, as evidenced by a large retrospective study evaluating 9991 IVF cycles by Wu et al. that found that increasing male age had no association with implantation rate when the female partner was in the < 30 year or 35–38 year categories, but interestingly implantation rate was decreased with increasing paternal age in the 30–34 female age group, though the authors suggest that this discrepancy may be due to small sample sizes in the other two groups [126].

#### Pregnancy rate

##### In IVF cycles

As with other outcomes, maternal age has an important role in the potential adverse effects of paternal age on pregnancy rate and therefore likely clouds many of the findings. Overall, one study found that pregnancy was highest when paternal age was < 30 years [126]. Similarly, a prospective study on 221 gamete intrafallopian transfer (GIFT) or IVF cycles showed a decrease in pregnancy rate with increasing male age [69]. However, other studies have found varying results based on maternal age group. de La Rochebrochard et al. found that the rate of pregnancy failure for cycles with paternal age ≥ 40 years was higher as maternal age increased [26]. In a retrospective study on 103 oocyte donation cycles, where maternal age seems to have no or minimal effect, no effect of APA (> 37 years) on pregnancy rate was observed [53].

##### In IVF/ICSI split cycles

In a recent study on whether male age is associated with the clinical outcomes of IVF/ICSI cycles for idiopathic infertility, the odds of clinical pregnancy were found to decrease by 3% for each year increase in male age after adjusting for female age [56]. Similarly a large study evaluating 1023 cycles using anonymous donor eggs to control for female age showed a significant increase in pregnancy loss in cycles with fathers > 50 years of age [42]. In contrast, Luna et al. reported that clinical pregnancy was not associated with APA even in men > 60 years and no significant relationship was found between APA and pregnancy [74].

##### In ICSI cycles

As with fertilization rate, data regarding ICSI cycle outcomes for pregnancy rate are not well-established and are difficult to evaluate due to the many potential confounders. Kaarouch et al. evaluated 83 IVF cycles using ICSI and found that rates of clinical pregnancy were lower and rates of embryo transfer cancellation were higher in older men (≥ 40 years) compared to men < 40 years old [63]. Similarly, in a retrospective study of 484 cycles using donor oocytes, male age was significantly lower in the group that became pregnant in comparison with the non-pregnant groups [48]. Several other studies, however, have not found any association between paternal age and pregnancy rate, including in donor oocytes [1, 7, 17]; notably, in one of these studies, a significant raw association was no longer significant after adjusting for confounding factors [17], highlighting the potential for unknown effects of confounders and modifiers to be contributing to the mixed results. To this end, two studies have accounting for oligozoospermia but also found mixed results. Paternal age has been found to have a negative correlation with pregnancy rate in oligozoospermic patients, with a decrease of 5% for every year in paternal age, but no association in normozoospermic patients [36]. In contrast, a study using testicular spermatozoa from azoospermic patients found that paternal age has no association with pregnancy rate (Tsai, Lan et al. 2013).

#### Live birth rate

Several studies have found significant decreases in rates of live birth associated with increasing paternal age, with one study on GIFT and IVF cycles finding a 12% decreased odds of live birth for every year of paternal age [69] and another study of 2425 cycles of couples with idiopathic infertility finding a 4.1% decreased odds of live birth for every year of paternal age after fully adjusting results for appropriate confounders. The worst outcomes were seen in men >50 compared to those < 40 years [56]. Similarly, in a study of 237 donor oocyte cycles, the authors found a 26% decreased odds of live birth for every 5 year increase in paternal age [100]. Other studies have found no associations between live birth rate and paternal age in azoospermic patients using cryopreserved testicular spermatozoa [116], in couples with male versus female factor infertility with fathers > 50 years old [92], and in a frozen donor oocyte model with a APA cut-off of > 35 years [17].

As with other outcomes, female age may be confounding or modifying this relationship. Female age determines the capacity of oocyte to repair sperm DNA damage [55]. When the quality of oocytes is not optimal due to AMA, the presence of any damage in the sperm genome results in fertilization failure, poor quality embryos, an increase in miscarriage, or a reduction in pregnancy and delivery rates. Several studies have found that, after adjusting for female age or when using donor oocytes, parental age had no association with live birth rate [7, 53, 87, 123]. Female partner plays a very important role in determining the detrimental effects of male aging on ART outcomes, and results from a retrospective study evaluating 4057 first cycles indicate that maternal factors may be modifying the effect of APA; although the associations between age and pregnancy rate were more notable for maternal age than for paternal age, the largest negative association was seen when both parents were of advanced age [81].

##### Miscarriage rate

Increased rates of miscarriage in older couples are shown in several studies, likely because sperm from older men have more DNA damage and oocytes from AMA women have lower capacity to repair sperm DNA damages [55]. Additionally, the increasing chromosomal abnormalities associated with APA may also be contributing to miscarriage rates [110]. Unfortunately, the data fail to provide a clear picture of the relationship between APA and miscarriage; this discrepancy may partially be due to different definitions of the cut-off for APA, differing study design, and whether donor or patient oocytes were used.

Fully adjusted results in couples with idiopathic infertility indicated a 4.5% increase in miscarriage rate for every increased year of male age [56]. Similarly, in a case-control study of 13,865 women, Kleinhaus et al. reported that the odds of spontaneous abortion of embryos with fathers ≥40 years was 60% greater than risk in the reference group (25–29 years) [68]. In fertile population, pregnancies conceived by men over 45 were 87% more likely to end in miscarriage than pregnancies conceived by men under 25 years, although when stratified by trimester, the effect of paternal age was only significant for first trimester miscarriage [110]. de La Rochebrochard and Thonneau, evaluated 3174 planned pregnancies in a population registry, and found that the odds of having a miscarriage was 80% greater when the paternal age was 40–64 years and maternal age is 20–29 years than in couples where both parents were 20–29 years; the group at highest risk for miscarriage were couples where the woman was ≥35 and the man ≥40 [27].

Despite these data, there have also been several studies that have not found a relationship between APA and miscarriage rate, including in a study with donor oocytes in ICSI cycles [7], a systematic review [22], in normozoospermic, oligozoospermic [36], and azoospermic patients [116], and in a large study of IVF cycles that stratified by paternal age [126]. Finally, Cito et al. also did not find any significant difference for miscarriage rate between men >45 years and ≤ 45 years undergoing ICSI cycles using donor eggs [21]. These disparities in findings are, as with other studies on paternal age effects, likely at least in part attributable to the simultaneous effects of maternal age and the collinearity of maternal and paternal age [89], which may be supported by the lack of an effect of sperm donor age on miscarriage found in a large study of over 46,000 women, though sperm donors likely do not well-represent the male population as a whole due the selection guidelines of most sperm banks [47].

### Offspring health

Finally, the combination of poorer sperm quality, DNA integrity, and ART outcomes likely has an effect on the longer-term outcomes of children born to these older fathers. Several of the outcomes that are associated with paternal age, such as leukemia and schizophrenia, likely have genetic components. Others, however, such as behavior and social function, may be more associated with social factors related to having older parents or those who choose to have children at a later age. Irrespective of their cause, understanding these potential long-term outcomes are essential to adequately counseling prospective fathers on the risks of having children at an advanced paternal age.

#### Perinatal outcomes

Although AMA is known to be associated with poorer birth outcomes, paternal age has also been associated with increased risk of stillbirth [3, 5, 78]; including in a large study from Denmark of all pregnancies there from 1994 to 2010, which found increasing risk of stillbirth with increasing age category compared to fathers 30–34 years old, men 50 years or older having a 58% chance greater risk of stillbirth [118]. APA has also been associated with low birth weight (LBW) [3, 49] and preterm and very preterm birth [3]; LBW was also associated with APA in couples with young maternal age and advanced paternal age [33]. However, other studies have found such poor outcomes only in children of teenaged fathers, with no increased risk with APA [20] or, in a large study of all births in Ohio from 2006 to 2012, no associations were found with paternal age at all, including preterm birth, growth restriction, and NICU admission [57]. In contrast, a large study of infant check-up data at one Japanese medical center found increased birth weight with advanced paternal age, but only in non-first-born children and growth parameters at 1 month of age were no longer associated with paternal age [59].

#### Overall health

Advanced paternal age has long been associated with several syndromes in offspring, most of which are associated with single DNA errors, such as achondroplasia and osteogenesis imperfecta, as well as neurofibromatosis and Marfan syndrome and caraniosynostoic diseases such as Apert’s, Pfeiffer’s and Crouzon’s syndromes. More recent research has found an association with more common disorders including diabetes mellitus [73, 91], obesity at young adulthood [34], and greater insulin sensitivity and increasing night time systolic and diastolic blood pressure [2]. Interestingly, APA (≥35 years) may be related to reduced risk of asthma in children [114]. Although a recent animal study showed that offspring born by aged fathers have shorter lifespan [127], human research has indicated that children of older fathers may live slightly longer than those of younger fathers [19]. However, due to increased risk of congenital anomalies, malignancies, and other causes, children under 5 of older fathers are at increased risk of death, with the risk in offspring of fathers aged 45 and over is 65% higher than those whose fathers were 30–34 at the time of their birth [117]. Offspring of fathers aged 45 or over have also been found to have an 18% higher odds of seizures as infants than offspring of fathers aged 25–34 years old [64]. Paternal age may also affect overall brain morphology, with one study finding increased cortical volume with age up to a paternal age of 30, and then a decline in cortical volume, most pronounced in cortical surface area [106].

#### Birth defects

APA has been associated with many forms of birth defects including a 26% increase in risk of musculoskeletal congenital anomalies in fathers ≥50 years in comparison with those 30–34 years [119]. A large US study of births from 1989 to 2002 found slight increased risk of limb, integument, and nervous system anomalies in fathers over 35 versus 29 years [51], a 5% increase in respiratory system anomalies for every 5 years of male age, and increased risk of eye, genital, upper limb, and other malformations in children of older fathers [51]. Findings on musculoskeletal anomalies were supported by a similar study in Denmark, which found a 6% increased risk of these anomalies for every 10 year increase in paternal age [119]. In contrast, risks of pyloric stenosis and great vein anomalies have been found to be decreased with every 5 years of paternal age, and no association was found between increasing paternal age and heart defects, neural tube defects, or oral clefts [51]. Oral cleft has been comparatively frequently studied in association with paternal age, with a study of over 2.4 million births in Norway from 1967 to 2010 finding no individual association between paternal or maternal age and oral clefts once the other parent’s age was included in the model, but increased risk in couples where both parents were of advanced age [11]. A metanalysis of 13 studies found a 58% increased risk of cleft palate in children of fathers over 40 in comparison with fathers aged 20–39 [28]. However, other large studies, including one of all Ohio births from 2006 to 2012, have not found any association between APA and congenital anomalies [57].

#### Childhood cancers

Leukemias are the most common causes of childhood cancers in children up to age 14, followed by brain and central nervous system tumors, and then lymphoma [108]. Several large studies have evaluated the risk of leukemia in the offspring of fathers of APA, with several finding increased risk of acute lymphoblastic leukemia (ALL) [31, 104] but no [95] or a protective [104] effect on risk of acute myeloid leukemia. A large meta-analysis of 11 case control and 5 nested case control studies including data from 1968 to 2015 found a 5% increased risk of ALL in the case control studies and 4% in the nested case control studies [96]. Infantile leukemia, in contrast, has been found to be associated with younger paternal age, but not APA [75]. Lymophoma has similarly been associated with APA, with a large US case-matched control study finding a 3% increased risk of pediatric cancers for every 5 years of paternal age [121]. A large study of Swedish patients with lymphoid neoplasms found that the association between increased paternal age and increased risk of these neoplasms held true even for patients diagnosed as late as in their 70s [71]. Data on CNS tumors, the second most common cause of cancer in children after blood cell cancers, is similarly mixed, with one study finding a 5% increased risk for every 5 years of paternal age, with an 8% increased risk of astrocytoma for every 5 years [90], and others finding no increased risk at all once other factors were included in the models [121]. A study of UK children born 1968–1986 found a 3 times greater risk of retinoblastoma in offspring of men ≥45, though this was not statistically significant.

#### Psychiatric disorders

Older paternal age has been associated with a range of psychiatric disorders including schizophrenia, autism spectrum disorder (ASD), mental disability, starting at least at age 45 [80], as well as increased risk of bipolar disorder. There is a moderate certainty of evidence [91] of increased risk of schizophrenia with increasing paternal age [89], starting at a paternal age as early as 25 years [40], including four meta-analyses [83, 91, 115, 124]. Wang et al. found that paternal age has an independent role in early onset of schizophrenia in offspring [122]. There is a weak evidence for association between paternal age and bipolar disorders [89], although findings have been inconclusive. A recent study evaluating 314 subjects found that paternal age is a gender-independent and non dose-dependent risk factor for bipolar disorder and also a risk factor for unipolar disorder [41].

Autism Spectrum Disorder (ASD) has similarly been linked to APA [89], with a 55% increase in ASD risk with APA and a 21% increased risk with each 10 year increase in male age, based on two meta-analyses (Wu, [91, 125]). Idring et al. evaluated 417,303 Swedish children and showed a direct correlation between ASD and paternal age [58]. The risk of ASD is 6.3 times more in offspring of older fathers (≥40 years) compared to younger fathers [15].

#### Behavior, development and social function

Poor social skills and function have been identified as predictors of later diagnosis of schizophrenia and have been identified in siblings of those with schizophrenia, and so have also been explored in relation to APA. Offspring of fathers 45 or older were more likely to have poor social functioning, including friendships, relationships, and social activities, than those with fathers of 25–29 years. In support of this, APA has also been associated with lower chance of marriage and higher rate of childlessness [37]. However, the poorer social skills discussed above may represent improved long-term achievement; in a study by Janecka et al. [60], the authors calculated a “geek index” which combined high IQ, strong focus on a subject of interest, and social aloofness, which was associated with APA and also better educational outcomes at age 16 and longer term academic achievement [60].

The role of APA in development and academic achievement has been inconclusive [102, 112], though, as several studies have found associations with APA and developmental delay such as Bayley Scales for Infant Development and educational attainment [43], with one study finding a 9% increase of offspring being classified as delayed for every 10 years of additional paternal age [88] and another study finding offspring of older fathers did more poorly on neurocognitive tasks [102]. These outcomes may only apply to younger children, though, as APA was not found to be associated with decreased grades in adolescents [79, 112] and offspring of fathers over 50 had slightly higher grades than their peers with younger fathers [112].

## Conclusions and future directions

Although the inverse relationship between pregnancy rate and age of the female partner has been well-established, there are few studies examining the relationships between paternal age and pregnancy and ART outcomes and offspring’ health. However, the current data implies that aging impairs sperm quality, which can affect the fertility status and ART outcomes. The complications of confounding factors, given that most APA fathers are partnered to AMA mothers, and the lack of systemic definitions and study design has contributed to difficulty in interpreting data; there is no clear definition of APA in the current literature. Based on the confounding factors identified in many of the studies discussed above, APA should be evaluated while also accounting for normal or abnormal semen parameters, age of female partner, and mode of conception.

One option to prevent adverse effects of APA on pregnancy outcome would be sperm cryopreservation at a young age, just as oocyte cryopreservation is discussed for female patients who intend to delay child-bearing until AMA. However, sperm cryopreservation itself may reduce sperm quality including reduced viability and motility and increased DNA fragmentation [4, 72]. These outcomes may offset the benefits of using “younger-aged” sperm, and there are insufficient data to know whether the benefits outweigh the risks at this time. Also new adjuncts to ART procedures like morphologically-selected sperm injection and evaluation for DNA fragmentation could be offered to older fathers. Doing pre-implantation genetic testing for embryos created using older fathers’ sperm or aneuploidy screening of sperm cells from men in advanced should also be considered. Using antioxidants and sperm motility enhancers such as pentoxifylline in vitro for sperm preparation and in vivo in mens’ diets or as supplements [4] have been found to be associated with improved sperm quality and may help improve outcomes in older fathers.

These solutions, although may address the sperm characteristics and health risks to offspring, do not address the effects of having older parents on child growth and lifestyle. As paternal age increases, increased consideration has been given to the ethics of advanced age at parenthood. In addition to health risks, children of older parents are more likely to experience early parental death, which has been associated with shorter life expectancy in offspring [86], and results in many of these children experiencing their adolescence and young adulthood without one or both of their parents. In one study that found increased risk of mortality in those with older parents, this risk was found to be mediated in part by parental survival until the offspring was at least 35 [86], highlighting the impact of losing parents at a young age. Having older parents also interferes with offspring’s ability to establish their own careers and families, as they often must take on a caregiving role earlier than their peers, which can impair their own happiness, socioeconomic status, and continue the cycle of older parenting [14]. These challenges must be considered on top of the more concrete challenges of fertility, physical health, and other risks discussed in this manuscript. Additional studies are needed to ensure that accurate information are provided to patients regarding the risks of APA, especially for older men who are seeking to father a child, and that all potential implications of APA are considered by physicians and patients.

## Data Availability

N/A

## Abbreviations

US: United Stated
ARTs: Assisted Reproductive Technologies
IUI: Intrauterine Insemination
IVF: In Vitro Fertilization
ICSI: Intracytoplasmic Sperm Injection
APA: Advanced Paternal Age
AMA: Advanced Maternal Age
mL: Milliliter
DNA: Deoxyribonucleic Acid
ASRM: American Society for Reproductive Medicine
HDS: High DNA Stainability
DFI: DNA Fragmentation Index
FISH: Fluorescent In Situ Hybridization
GIFT: Gamete Intrafallopian Transfer
LBW: Low Birth Weight
NICU: Neonatal Intensive Care Unit
ALL: Acute Lymphocytic Leukemia
CNS: Central Nervous System
UK: United Kingdom
ASD: Autism Spectrum Disorder
IQ: Intelligence Quotient
